# Anterior chamber paracentesis after central retinal artery occlusion: a tenable therapy?

**DOI:** 10.1186/1471-2415-14-28

**Published:** 2014-03-10

**Authors:** Achim Fieß, Ömer Cal, Stephan Kehrein, Sven Halstenberg, Inez Frisch, Ulrich Helmut Steinhorst

**Affiliations:** 1 Department of Ophthalmology, Dr. Horst Schmidt Clinics Wiesbaden, Wiesbaden, Germany; 2 Center of Ophthalmology, Ingelheim, Germany

**Keywords:** Central retinal artery occlusion, Intraocular pressure, Paracentesis, Retinal vascular occlusion, Treatment

## Abstract

**Background:**

The goal of this study was to investigate the visual outcome of acute central retinal artery occlusion (CRAO) after current standard therapy with and without paracentesis. In addition, we investigated whether there was a dependence of the resulting visual acuity on the time between first symptoms and implementation of paracentesis. Finally, we analysed risk factors for CRAO.

**Methods:**

We performed a retrospective analysis of data from patients with CRAO who received standard in-patient therapy with and without paracentesis at the Dr. Horst Schmidt Clinics in Wiesbaden, Germany between 2000 and 2012. The primary endpoint was the change of visual acuity 3 days after the initiation of intervention.

**Results:**

Data from 74 patients with CRAO were included in the study. Fifteen patients were treated conservatively and 59 patients received additional paracentesis. Clinically significant improvement of BCVA (logMAR ≥ 0.3) after 3 days was observed in 26.7% of patients without paracentesis, 36.4% of patients with paracentesis within 6 hours, 20% of patients with paracentesis within 7–24 hours, and 23.1% of patients with paracentesis more than 24 hours after the onset of symptoms. There was no significant difference in the outcome between patients with (BCVA 1.9 ± 0.31) and without paracentesis (BCVA 1.75 ± 0.32) (p = 0.9), nor among the groups with paracentesis (p = 0.8). One patient suffered a lens injury due to the paracentesis, with subsequent need for cataract surgery.

**Conclusions:**

There was no added gain in visual acuity by performing a paracentesis, independent of the time elapsed between first symptoms and the implementation of paracentesis. In the absence of any tangible effectiveness of paracentesis and the inherent risks of paracentesis such as intraocular infection and injury, paracentesis does not appear to be warranted as a treatment of CRAO.

## Background

Acute central retinal artery occlusion (CRAO) is an ophthalmological emergency caused by closure of the central retinal artery by a thrombus or embolus [1,2]. Clinically, the patient notices a sudden and painless unilateral loss of vision. Even if there is only a short closure of the central retinal artery, CRAO leads to permanent ischaemic damage of the retina. Therapy approaches described in the medical literature include systemic anticoagulation [3], systemic venous thrombolysis [4,5], catheter-guided intra-arterial fibrinolysis [3], ocular massage [2,3] and reduction of intraocular pressure [2,6,7].

Despite the numerous therapeutic approaches there is no effective therapy to date that restores function of the retina to a satisfying extent. Reduction of intraocular pressure to improve intraocular blood flow is frequently discussed as a possible treatment option. Approaches toward lowering intraocular pressure include administration of systemic [7,8] and local [3] medications, ocular massage [2,3], and paracentesis [6,9].

Several studies have shown that the retina only has a very short tolerance for ischaemia, which was determined to be 105 minutes in animal experiments [10-12]. If ischaemic conditions persist longer than this period, permanent retinal damage seems inevitable. Since the extent of retinal damage is a function of the duration of ischaemia, it is important that a countermeasure against CRAO be effective quickly and available at any time.

Paracentesis leads to a rapid reduction of intraocular pressure, and therefore is believed to promote retinal perfusion, especially in the first hours after CRAO [7,9]. Advantages of paracentesis are that the implementation is quick and involves low costs and few resources. On the other hand, paracentesis poses a considerable risk of infection.

Most studies regarding the medical benefit of paracentesis examined only whether there is a gain in visual acuity for patients with CRAO after paracentesis, but did not consider the time between first symptoms and the initiation of therapy [9]. In the present study we investigated whether paracentesis improves visual acuity after CRAO depending on the time between first symptoms of CRAO and the implementation of paracentesis. Furthermore, we analysed the predictive value that frequent risk factors for CRAO might have. These risk factors include arterial hypertension [13,14], hypercholesterolaemia [15], atherosclerosis [16], afflictions of the carotid arteries [17], cardiac arrhythmias [15], and diabetes mellitus [18]. Overall, this study aims to address the question of whether the benefits of paracentesis justify the associated risks.

## Methods

**Figure 1:**
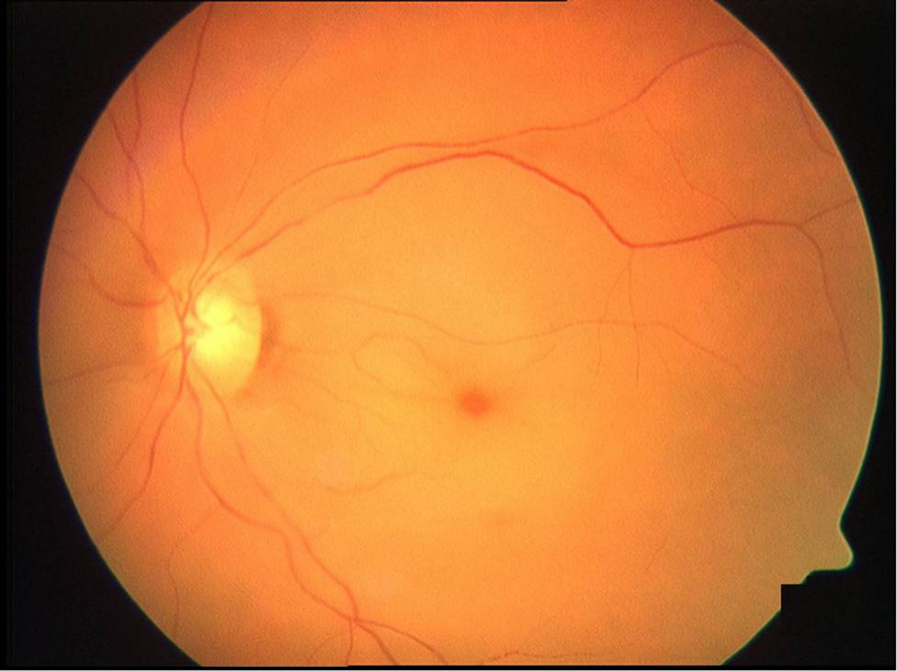
Typical central retinal artery occlusion with cherry-red spot, retinal oedema and narrowing of the vessels.

We performed a retrospective analysis of data from patients with CRAO treated between 2000 and 2012 with standard in-patient therapy with or without paracentesis. All patients received the same conservative therapy and the group of patients with paracentesis received additionally a paracentesis that was conducted in addition to the conservative treatment after the diagnosis was found. Diagnosis was based on funduscopic evaluation by the treating ophthalmologist and positive identification of typical CRAO characteristics such as a cherry-red spot in the macula and a reduction in arterial blood flow (Figure 1).

Arteritic aetiology was excluded by the absence of elevated inflammatory parameters and verification of the typical clinical presentation of CRAO. In the present study we only included patients with non-arteritic CRAO with thromboembolic origin. Arteritic, transient ischaemia and CRAO with a preserved cilioretinal vessel were excluded. Additionally, the time between first symptoms and implementation of the paracentesis had to be traceable. All patients were treated in an in-patient setting. Standard treatment consisted of routine anticoagulation with low molecular weight heparin adjusted to patient body weight twice daily, bloodletting for hematocrit ≥40%, and paracentesis in certain cases.

Paracentesis included local anaesthesia with proxymetacaine hydrochloride, application of prophylactic antibiotic eye drops, and a brief puncture of the cornea with a paracentesis blade to drain a few drops of aqueous fluid. Successful execution was verified with an applanatory intraocular pressure measurement that had to be under 5 mmHg. In case of contraindications or a decline by the patient, paracentesis was waived.

Best corrected visual acuity (BCVA) was measured at the beginning of treatment and after 3 days. In addition, we recorded current medication, demographic data, the onset of initial CRAO symptoms, and the time of paracentesis. Furthermore, an analysis of cardiovascular risk factors was performed and the test results were documented. In particular, blood pressure was measured routinely four times during the first 24 hours. The average of these measurements was used for the statistical analysis of the blood pressure at the beginning of therapy. Finally, any complications associated with paracentesis were documented.

The data collection in this study has been performed in accordance with the Declaration of Helsinki. Because of the retrospectivity of the study and complete anonymisation of patient data, ethics committee approval was not required [19]. The data collection complied with all applicable institutional, national and international guidelines.

### Data analysis and end points

The primary endpoints were the change in visual acuity 3 days after the beginning of treatment and the time between first symptoms and initiation of therapy.

Visual acuity was determined on the basis of numbered optotypes and converted for the analysis into the logarithm of the minimum angle of resolution (logMAR). This was carried out for all visual acuities ≥1/250. If visual acuity was less than 1/250, the following logMAR values were assigned: counting fingers = 1.9, hand motion = 2.0, light perception = 2.1, and no light perception = 3.0.

A significant clinical improvement in visual acuity was defined based on the EAGLE Study [20] as improvement in visual acuity of at least 0.3 log units. Secondary endpoints were any complications associated with the paracentesis and the patients’ cardiovascular risk factors.

### Statistical analysis

The assumption of a normal distribution of data points was tested by the Kolmogorov-Smirnov test. The Chi-square test was used for the statistical comparison of nominal parameters. The Mann–Whitney U test was used to compare groups with or without paracentesis. The Kruskal-Wallis H test was used to compare the different groups receiving paracentesis. All results were expressed as mean ± standard deviation, except where otherwise specified. A value of p < 0.05 was considered statistically significant. The incorporation of cardiovascular risk factors into the statistical analysis was merely descriptive.

## Results

This study includes data from 74 patients with CRAO treated at the Dr. Horst Schmidt Clinics in Wiesbaden between 2000 and 2012. The patients included 39 males and 35 females. The mean patient age was 73.9 ± 9.8 years. Fifteen patients were treated conservatively and 59 patients received additional paracentesis. In patients receiving paracentesis, the procedure was performed in 11 patients (18.6%) within 6 hours, 35 patients (59.4%) within 7–24 hours, and 13 patients (22.0%) beyond 24 hours after first symptoms (Table 1).

**Table 1:** Overview of patient pool, study parameters, and evaluation times

	**Paracentesis**				**No paracentesis**	
	**≤6 h**	**7–24 h**	**>24 h**	**all pat.**	**all pat.**	**p**
Total number of patients [n]	11	35	13	59	15	
Male [n (%)]	7 (63.6%)	18 (51.4%)	9 (69.2%)	34 (57.6%)	5 (33.3%)	0.1
Right Eye [n (%)]	6 (54.5%)	15 (42.9%)	5 (38.5%)	26 (44.1%)	8 (53.3%)	0.5
Age [years]	72.3 ± 13.8	73.5 ± 8.6	73.6 ± 11.9	73.3 ± 10.3	76.7 ± 7.6	0.2
Eye pressure at admission [mmHg]	13.6 ± 3.0	15.1 ± 3.1	16.1 ± 8.7	15 ± 4.8	13.3 ± 3.5	0.2
Time to treatment [h]	4.1 ± 1.2	15.3 ± 6.3	43.9 ± 4.4	28.2 ± 37.7	24.4 ± 16.0	0.3
Length of hospital stay [days]	4.6 ± 1.6	3.9 ± 2.1	3.9 ± 1.7	4.1 ± 1.5	4.9 ± 2.7	0.4
Bloodletting performed [n (%)]	2 (18.2%)	19 (54.3%)	5 (38.5%)	26 (44.1%)	4 (26.7%)	0.2

The p-values indicate no significant differences between patients treated with or without paracentesis.

### Starting visual acuity

The BCVA [logMAR] at the start of standard treatment in the group of patients without paracentesis was 1.92 ± 0.21. The BCVA was 1.96 ± 0.16 for patients with paracentesis within 6 hours, 1.99 ± 0.25 for patients with paracentesis within 7–24 hours, and 2.05 ± 0.32 for patients with paracentesis more than 24 hours after first symptoms (Table 2). There was no significant difference between the groups with and without paracentesis (p = 0.4), nor between groups of patients with paracentesis at different times after onset of CRAO (p = 0.9).

**Table 2:** Visual acuity at the start of therapy and after 3 days

	**Paracentesis**				**No paracentesis**	
	**≤6 h**	**7–24 h**	**>24 h**	**all pat.**	**all pat.**	**p**
Baseline visual acuity [logMAR]	1.96 ± 0.16	1.99 ± 0.25	2.05 ± 0.32	2 ± 0.25	1.92 ± 0.21	0.4
Visual acuity after 3 days [logMAR]	1.78 ± 0.21	1.92 ± 0.3	1.94 ± 0.39	1.9 ± 0.31	1.75 ± 0.32	0.3
Gain of visual acuity [log units]	−0.18 ± 0.21	−0.07 ± 0.15	−0.11 ± 0.19	−0.1 ± 0.17	−0.17 ± 0.33	0.9
*Visual acuity after 3 days:*						
-clinically better [n(%)]	4 (36.4%)	7 (20.0%)	3 (23.1%)	14 (23.7%)	4 (26.7%)	
-clinically equal [n(%)]	7 (63.6%)	27 (77.1%)	10 (76.9%)	44 (74.6%)	11 (73.3%)	0.9
-clinically worse [n(%)]	0 (0%)	1 (2.9%)	0 (0%)	1 (1.7%)	0 (0%)	

The p-values indicate no significant difference in the outcome between all patients with and without paracentesis. There was also no significant difference between the groups of patients with paracentesis at different times after onset of CRAO.

### Change in visual acuity and outcome

Changes in visual acuity and outcome are shown in Table 2. Three days after initiation of treatment the BCVA was 1.75 ± 0.32 in patients without paracentesis. In patients that received paracentesis within 6 hours after first symptoms the BCVA was 1.78 ± 0.21 after three days of our treatment. In the group of patients with paracentesis within 7–24 hours after the onset of CRAO the BCVA was 1.92 ± 0.3 after three days, and in the group of patients with paracentesis more than 24 hours after first symptoms the BCVA was 1.94 ± 0.39 after three days. There was no significant difference in the BCVA after three days between patients with and without paracentesis (p = 0.3) nor between groups of patients receiving paracentesis at different times after CRAO (p = 0.2).

**Figure 2:**
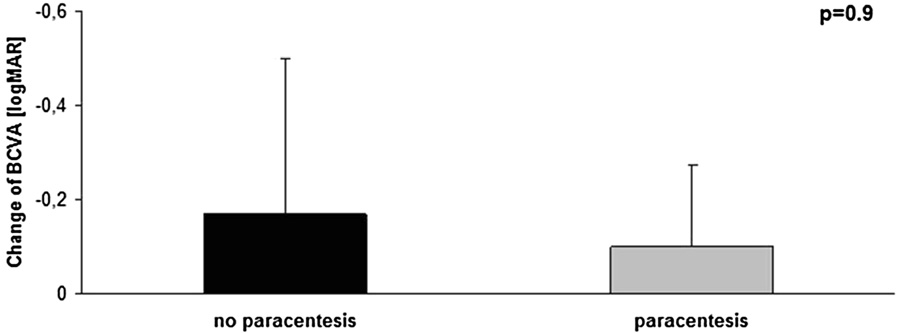
**Average change in visual acuity from time of hospital admission to three days after initiation of treatment for CRAO.** The p-value of 0.9 indicates no significant difference with respect to vision improvement between patients treated with or without additional paracentesis.

The improvement in BCVA after 3 days was −0.17 ± 0.33 in patients without paracentesis and −0.1 ± 0.17 in patients with paracentesis (Figure 2). There was no significant difference between patients with and without paracentesis (p = 0.9). The improvement was −0.18 ± 0.21 in patients treated with paracentesis within 6 hours after the first symptoms, −0.07 ± 0.15 in patients with paracentesis within 7–24 hours, and −0.11 ± 0.19 in patients with paracentesis 24 hours after first symptoms. There was no significant difference among the different groups of patients that underwent paracentesis (p = 0.2).

A clinically significant improvement of BCVA (logMAR ≥ 0.3) after 3 days was observed in 26.7% of patients without paracentesis, 36.4% of patients treated with paracentesis within 6 hours, 20.0% of patients who underwent paracentesis within 7–24 hours, and 23.1% of patients who received paracentesis more than 24 hours after the onset of symptoms (Table 2). There was no significant difference in the outcome between patients with and without paracentesis (p = 0.9), nor among the different groups treated with paracentesis (p = 0.8).

In 53.4% of patients without paracentesis the visual acuity after 3 days of conservative treatment was only hand motion or worse. A visual acuity of hand motion or worse was observed in 36.4% of patients after implementation of paracentesis within 6 hours of first CRAO symptoms, in 65.7% of patients in the group treated with paracentesis within 7–24 hours, and in 61.6% of patients in the group that underwent paracentesis 24 hours after first symptoms.

### Dependence of subsequent visual acuity on the time between first CRAO symptoms and initiation of treatment

**Figure 3:**
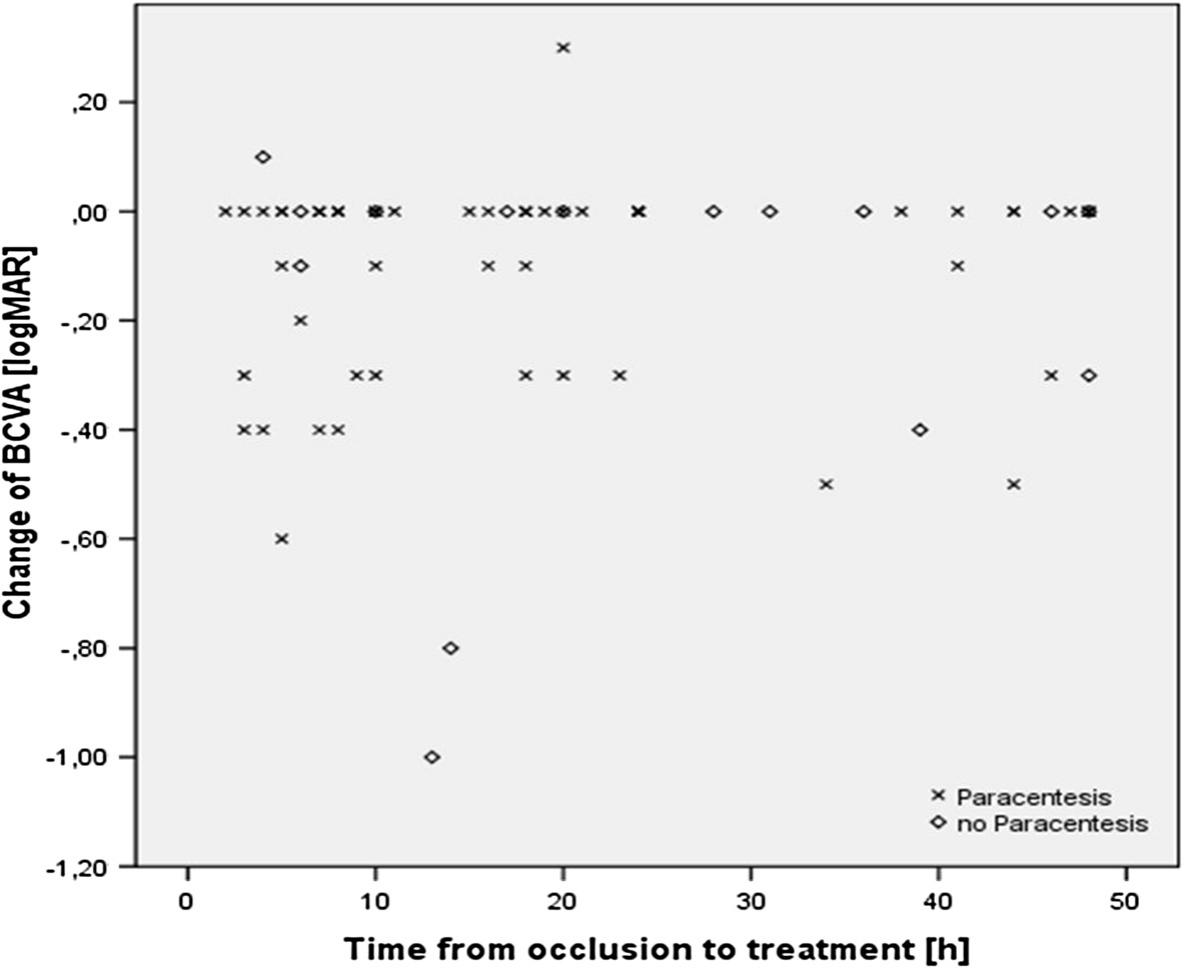
**Change in visual acuity for patients from time of hospital admission to three days after initiation of CRAO treatment as a function of the time elapsed between first symptoms and the initiation of therapy.** No detectable functional dependence existed between these two parameters for the group of patients with conservative treatment (p = 0.8) and in the group treated with paracentesis (p = 0.4).

Based on our results, the time elapsed between first symptoms of CRAO and the start of therapy had no significant influence on the outcome of visual acuity in the group of patients with conservative treatment (p = 0.8) or in the group treated with paracentesis (p = 0.4) (Figure 3).

### Thromboembolic risk factors

The thromboembolic risk factors recorded upon hospitalisation are shown in Table 3. Arterial hypertension was the most common risk factor. A history of arterial hypertension was present in 81.1% of patients at admission. In general, systolic blood pressure during the first 24 hours after admission was very high. Only 23.0% of patients had systolic blood pressure values in the normal range (120–139 mmHg), 48.6% had grade 1 hypertension (140–159 mmHg), 18.9% had grade 2 hypertension (160–179 mmHg), and 9.5% had grade 3 hypertension (≥ 180 mmHg).

**Table 3:** Cardiovascular risk factors among patients with CRAO in this study

	**Paracentesis**				**No paracentesis**	
	**≤ 6 h**	**7–24 h**	**>24 h**	**all pat.**	**all pat.**	**p**
	**(%)**	**(%)**	**(%)**	**(%)**	**(%)**	
Arterial hypertension	81.8	82.9	76.9	81.4	80.0	0.9
Hyperlipidaemia	36.4	20.0	23.1	23.7	13.3	0.6
Diabetes mellitus	9.1	14.3	15.4	13.6	33.3	0.1
Atrial fibrillation	9.1	2.9	7.7	5.1	20.0	0.1
CHD/valvular heart disease	9.1	28.6	15.4	22.0	20.0	0.9
History of embolic event	18.2	11.4	15.4	13.6	26.7	0.2

The p-values indicate that the distribution of risk factors was random and evenly distributed among the patients with and without paracentesis.

Overall, a haematocrit ≥ 40% was documented in 40.5% of all patients, with subsequent implementation of isovolaemic haemodilution, 20.3% of patients had a personal history of hyperlipidaemia, 8.1% suffered from atrial fibrillation, 17.6% had a history of diabetes mellitus, and 16.2% reported a previous thromboembolic event. Coronary heart disease or heart valve disease was documented in 21.6% of patients. No cardiovascular risk factors were reported by 16.4% of patients. An increased intraocular pressure (applanatory) above 21 mmHg was measured in 4.1% of patients. At the time of CRAO, 39.2% of patients were taking acetylsalicylic acid, and 4.1% were taking phenprocoumon.

### Complications of paracentesis

Paracentesis was performed without complications in 58 of 59 cases. One patient suffered a lens injury due to the paracentesis, with subsequent need for cataract surgery. None of the paracenteses resulted in intraocular infection.

## Discussion

The present study reveals no significant benefit of paracentesis to visual acuity after CRAO regardless of the time between first symptoms and initiation of therapy. This agrees with previously published data [9], where the gain in visual acuity was analysed independently of the time between CRAO and the onset of treatment. Moreover, in one case paracentesis led to lens injury requiring subsequent cataract surgery. These observations raise questions about paracentesis as a tenable treatment option for patients with CRAO.

One long-standing argument in favour of paracentesis is that lowering the intraocular pressure leads to a relative rise in the retinal perfusion pressure, possibly resulting in improved retinal blood flow. Implementing paracentesis and draining ocular fluid offers a quick and effective means to lower the intraocular pressure. In addition, this procedure is inexpensive and can be performed readily by any ophthalmologist with minimal preparation. On the other hand, an argument against paracentesis is the possibility of injuries to eye structures of the anterior segment as well as the risk of infections. In previous studies a gain in visual acuity due to paracentesis could not be proven [6,9]. Experiments showed that a decrease in intraocular pressure from 15 mmHg to 5 mmHg resulted in a relative increase of the perfusion pressure of only 15% and an increase of arterial blood flow of only 20% [7,9].

As a result of the retina’s low ischaemic tolerance, irreversible retinal damage occurs shortly after arterial occlusion [10-12]. Based on clinical data, it is assumed that visual acuity can recover partially up to 48 hours after blood vessel closure [21]. Schumacher *et al.*[3] showed a relationship between the eventual visual acuity and the time from arterial occlusion to the initiation of treatment. The study concluded that a delay in treatment within the first 20 hours after CRAO resulted in an irreversible loss of visual acuity of 0.2 lines per hour delay.

Contrary to this study, our data revealed no improvement of mean visual acuity in patients undergoing paracentesis within 6 hours of the first symptoms of CRAO relative to those undergoing paracentesis 6–24 hours or longer after the first symptoms. In agreement with the present data, Augsburger *et al.*[6] found that only 35% of patients with persistent CRAO showed a clinically significant improvement of visual acuity of at least three lines. Moreover, only 8% of CRAO patients showed an improvement of visual acuity of more than 0.1 [22,23].

In 92% of patients without therapy, the visual acuity after CRAO is counting fingers or worse [24]. Hayreh and colleagues [24] demonstrated that any possible increase of visual acuity after CRAO usually occurs within the first week or within the first few days after the occlusion. It was shown, that a clinically significant increase in visual acuity after weeks is rare [25]. Therefore, it is unlikely in our study that any significant relative improvement of visual acuity might have occurred beyond our follow-up time of 3 days among the patients treated with paracentesis. The most obvious explanation for the low visual acuity after CRAO is that current therapies do not yield the desired therapeutic success, and to date no sufficient therapy is available. The evaluation of possible therapies for CRAO is difficult because the effects of possible treatment options were often tested only in non-randomised and/or retrospective interventional studies. Previous studies served as the only reference.

Due to the low incidence of CRAO, which is only about 8.5 per 100,000 [2], there is also the problem of small case numbers for any randomised interventional study. The EAGLE Study [20] achieved a recruitment of only 84 patients over a period of 5 years. In two of the largest studies patients were included over a 30 year period [26,27]. The present study spanned 13 years.

Limitations of the study are a potential selection bias, short Follow-Up and the lack of angiographic examination after three days to evaluate whether the paracentesis improved retinal arterial perfusion even without visual improvement.

With no effective treatment for CRAO available it is important to identify the causes and focus on preventive measures. Cardiovascular mortality rate of affected patients was also shown to increase when risk factors for CRAO were ignored [28]. Therefore, diagnosis and adequate treatment of cardiovascular risk factors is of utmost importance. In 64–82% of patients with CRAO at least one previously undetected cardiovascular risk factor was diagnosed in the wake of a thromboembolic risk check-up [29]. Furthermore, 54% of patients with CRAO were under inadequate or no treatment of arterial hypertension, or suffered from a previously undetected, haemodynamically significant carotid stenosis [29]. This is consistent with our observation that systolic blood pressure during the first 24 hours of hospitalisation was significantly elevated in 77% of CRAO patients. Since it is widely thought that hypertension is a major risk factor for CRAO and other serious cardiovascular conditions, there is ample and compelling reason from an opthalmological point of view to control blood pressure tightly and aggressively.

Antiplatelet agents are used in neurology for protection against intracranial embolism. Accordingly, patients with CRAO are expected to benefit from such medications and avoid CRAO in the unaffected eye. However, the present study revealed that CRAO occurred in almost half of all patients taking the anticoagulants acetylsalicylic acid or phenprocoumon.

## Conclusion

We conclude that paracentesis is not recommendable as a therapy for CRAO at any time point after the arterial occlusion. This is based on the absence of any statistically significant improvement of visual acuity over conservative treatments, and the risks of endophthalmitis and injury to structures of the anterior segment associated with paracentesis. Due to the difficulties in preventing and treating CRAO reliably, this ophthalmological emergency continues to be an unpredictable and tragic event for anyone affected by it. Therefore, special emphasis and care should be dedicated to the interdisciplinary collaboration between general practitioner and ophthalmologist following a CRAO. The primary goal must be a comprehensive investigation of a patient’s individual risk factors for CRAO and their subsequent treatment. This should contribute to reducing mortality after CRAO and prevent the occurrence of CRAO in the unaffected eye.

## Abbreviations

BCVA: Best corrected visual acuity; CRAO: Central retinal artery occlusion; pat.: Patients.

## Competing interests

The authors declare that they have no competing interests.

## Authors’ contributions

AF has made substantial contributions to conception and design, acquisition of data, analysis and interpretation of data; and he has been involved in drafting the manuscript and revising it critically for important intellectual content; he has given final approval of the version to be published. ÖC, SK and SH have made substantial contributions to the article, acquisition of data, analysis and interpretation of data; and they have been involved in drafting the manuscript or revising it critically for important intellectual content; they have given final approval of the version to be published. IF has been involved in drafting the manuscript and revising it critically and has given final approval of the version to be published. UHS has been involved in drafting the manuscript and revising it critically and has given final approval of the version to be published. All authors read and approved the final manuscript.

## Pre-publication history

The pre-publication history for this paper can be accessed here:

http://www.biomedcentral.com/1471-2415/14/28/prepub
